# Retraction: Differential associations of non-chronic and chronic pain with dementia risk: insights from a cohort study

**DOI:** 10.3389/fnins.2026.2023236

**Published:** 2026-10-15

**Authors:** Frontiers Editorial Office

The journal retracts the 16 February 2026 article cited above.

Following publication, the authors alerted us to serious compliance concerns relating to the use and reporting of UK Biobank data in this article. Unauthorised data use is a breach of Frontiers' guidelines and those of the Committee on Publication Ethics. As such, this article is being retracted.

This retraction was approved by the Chief Executive Editor of Frontiers. The authors agree to this retraction.

